# “Well, not me, but other women do not register because...”- Barriers to seeking antenatal care in the context of prevention of mother-to-child transmission of HIV among Zimbabwean women: a mixed-methods study

**DOI:** 10.1186/s12884-018-1898-7

**Published:** 2018-06-28

**Authors:** Euphemia L. Sibanda, Sarah Bernays, Ian V. D. Weller, James G. Hakim, Frances M. Cowan

**Affiliations:** 1 Centre for Sexual Health and HIV/AIDS Research (CeSHHAR) Zimbabwe, 9 Monmouth Rd, Avondale West, Harare, Zimbabwe; 2 Department of International Public Health, Liverpool School of Tropical Medicine, Liverpool, UK; 3 Department of Health Services Research and Policy, London School of Hygiene and Tropical Medicine, London, UK; 4 Department of Infection and Population Health, University College London, London, UK; 5 Department of Medicine, University of Zimbabwe College of Health Sciences, Harare, Zimbabwe

**Keywords:** Antenatal care; PMTCT, Qualitative study, User fees, HIV testing

## Abstract

**Background:**

While barriers to uptake of antenatal care (ANC) among pregnant women have been explored, much less is known about how integrating prevention of mother-to-child transmission (PMTCT) programmes within ANC services affects uptake. We explored barriers to uptake of integrated ANC services in a poor Zimbabwean community.

**Methods:**

A cross-sectional survey was conducted among post-natal women at Mbare Clinic, Harare, between September 2010 and February 2011. Collected data included participant characteristics and ANC uptake. Logistic regression was conducted to determine factors associated with ANC registration. In-depth interviews were held with the first 21 survey participants who either did not register or registered after twenty-four weeks gestation to explore barriers. Interviews were analysed thematically.

**Results:**

Two hundred and ninety-nine participants (mean age 26.1 years) were surveyed. They came from ultra-poor households, with mean household income of US$181. Only 229 (76.6%) had registered for ANC, at a mean gestation of 29.5 weeks. In multivariable analysis, household income was positively associated with ANC registration, odds ratio (OR) for a $10-increase in household income 1.02 (95% confidence interval, CI, 1.0–1.04), as was education which interacted with having planned the pregnancy (OR for planned pregnancy with completed ordinary level education 3.27 (95%CI 1.55–6.70). Divorced women were less likely to register than married women, OR 0.20 (95%CI 0.07–0.58). In the qualitative study, barriers to either ANC or PMTCT services limited uptake of integrated services. Women understood the importance of integrated services for PMTCT purposes and theirs and the babies’ health and appeared unable to admit to barriers which they deemed “stupid/irresponsible”, namely fear of HIV testing and disrespectful treatment by nurses. They represented these commonly recurring barriers as challenges that “other women” faced. The major proffered personal barrier was unaffordability of user fees, which was sometimes compounded by unsupportive husbands who were the breadwinners.

**Conclusion:**

Women who delayed/did not register were aware of the importance of ANC and PMTCT but were either unable to afford or afraid to register. Addressing the identified challenges will not only be important for integrated PMTCT/ANC services but will also provide a model for dealing with challenges as countries scale up ‘treat all’ approaches.

**Electronic supplementary material:**

The online version of this article (10.1186/s12884-018-1898-7) contains supplementary material, which is available to authorized users.

## Background

Antenatal care (ANC) is important for ensuring the health of mothers and their babies [1, 2]. In the HIV era, ANC is a critical entry point for provision of prevention of mother to child transmission of HIV (PMTCT) interventions among HIV positive women. WHO recommends that over the course of pregnancy, all pregnant women should have at least four ANC assessments, with at least one during each of the following time points: 8–12 weeks, 24–26 weeks, 32 weeks and 36–38 weeks [3, 4]. Although three quarters of women are attended by a skilled health worker at least once during pregnancy [5], globally only half of women receive the recommended four ANC assessments, with estimates of 37% in countries with the highest rates of maternal mortality and 65% in Zimbabwe [6].

Many qualitative studies have been conducted in low and middle income countries (LMIC) to explore barriers to ANC uptake, both for the initial visit and for subsequent retention in care [7]. In a 2013 systematic review of qualitative studies, authors concluded that theories that underpin ANC programmes were at odds with cultural beliefs on pregnancy and the value of ANC, and that there are health system factors such as ANC costs, unavailability of resources at health facilities and poor staff attitudes that limit ANC uptake [7]. As has been recommended by WHO [8], many ANC programmes offer integrated HIV and PMTCT services. There is a dearth of literature exploring how integrating PMTCT with ANC affects these attitudes and barriers in LMIC. We aimed to quantitatively determine factors associated with ANC registration in the context of Zimbabwe’s national PMTCT programme and to qualitatively explore how some of the factors acted as barriers to seeking ANC among post-natal Zimbabwean women. This analysis was set in a wider study that was aimed at determining barriers to service uptake along various parts of the PMTCT cascade (the PMTCT cascade is a series of events/services that must be taken up by a woman to prevent mother-to-child transmission of HIV) [9, 10].

## Methods

### Description of study setting

The study was conducted at Mbare Clinic, located in one of the poorest townships (densely-populated suburbs) in Harare, Zimbabwe. The clinic is owned and run by the Harare City Health Department. National guidelines state that ANC should begin in the first trimester. At the time of this study, women were asked to pay a fixed fee of US$30 at the time of registering for ANC, which covered the cost of all examinations and tests conducted during pregnancy and delivery, the delivery itself, plus all postnatal care up to 6 weeks post-delivery.

### Study procedures

Between September 2010 and February 2011, a cross-sectional survey was conducted among post-natal women who were either enrolled into the study at time of delivery or when they attended for the 10-day postnatal visit. Recruitment was only conducted on weekdays during office hours. Women completed an interviewer-administered questionnaire if they met the following inclusion criteria: 1) had given birth up to 6 weeks prior to the interview, 2) resided in Mbare suburb and 3) were willing and able to give written informed consent (assisted by an independent witness if illiterate). The pre-tested paper-based questionnaire was administered by a female study nurse in the local language, Shona. The nurse had been trained on questionnaire completion, research ethics and good clinical research practises. Data collection was done in private at the clinic and participants were asked about their socio-demographic and clinical characteristics such as HIV status and obstetric history, income, (*Approximately how much money do you earn/source each month in US dollars?*), partners income (*Approximately how much money does your partner earn/source each month in US dollars?*) and food security (*In the last week, has an adult in your house skipped a meal or eaten less in order for there to be enough food for the children?* And *In the last week, have you had to go an entire day without eating because there was no food in your household?).* In addition, women were asked about the care they received during pregnancy, particularly whether they sought ANC (*Did you seek antenatal care after you discovered you were pregnant?*) the timing of ANC *(When did you first seek antenatal care?)* and HIV testing *(Have you been tested for HIV? Yes/No.* and *When were you tested for HIV?)*. This information was cross-checked with patient-held medical records, which were used to confirm date of registration and HIV status (Please see Additional file 1 for the full questionnaire).

For data analysis, a composite food security variable was created using the two food security questions above; a participant was food secure if she gave a negative response to both questions. Household income was calculated by combining participant and partner incomes. Data analysis was done using Stata Version 10. Chi-squared tests of association were run before univariable and multivariable logistic regression was done to determine factors associated with ANC registration. An explanatory variable was eligible for inclusion in the final model if there was evidence of its association with ANC registration at the *p* < 0.1 level. A causal diagram was used to determine which variables to include in multivariable analysis. Care was taken not to include collinear variables in the same model (variables that related to income and economic/food stability). Multivariable logistic regression was done by adding each variable to the model in turn, and using likelihood ratio tests to determine model fit. Investigations for effect modification, using likelihood ratio tests, were done between the following variables: 1) education and marital status, 2) marital status and whether pregnancy was planned, 3) marital status and household income.

In addition, the first 21 survey participants who had either not registered or had registered for ANC later than 24 weeks of pregnancy were interviewed in-depth by a trained social science researcher (ELS) to explore barriers to ANC. Interviews were conducted in participant’s language according to an interview guide that sought views on importance of ANC, reason for delayed or non-registration for ANC, experiences at home or clinic during the antenatal period and recommendations for how ANC uptake can be improved. Interviews lasted about forty-five minutes and were digitally-recorded, transcribed and translated into English by trained research assistants. Analysis was done by author ELS and began as soon as in-depth interviews started: field notes were written with attention to emerging themes, followed by interview summaries which compared thematic findings across interviews. A coding framework was then developed, incorporating both descriptive and analytic themes from all the interviews. Coding of each interview transcript was done by one researcher (ELS) according to this framework. Qualitative data were handled and analysed using NVIVO 10 (qualitative data management software).

### Ethical considerations

All participants gave written informed consent before participating. The study received ethical approval from Medical Research Council of Zimbabwe (ref MRCZ/A/1544) and University College London Research Ethics Committee (ref 2118/001).

## Results

### Cross-sectional survey results

#### Participant characteristics

Two hundred and ninety-nine women (54% of those eligible) completed the questionnaire. Their mean age was 26.1 years (95%CI 25.4–26.7). The women were very poor: only 30% earned their own income (Table 1), with an average monthly income of US$33.76 (95%CI $25.08–$42.45). Overall 89.3% were married and 91.8% of partners earned an income with a mean monthly income of $165.86 (95%CI $149.36–$182.35), giving a mean household income of $180.89 (95%CI $161.74–$200.03). The mean number of children per woman was 2.27 (95% confidence interval 2.13–2.42).

**Table 1:** Participant characteristics and associations with ANC registration

Characteristics	*N* (%)	n/N (%) Registered for ANC	Odds ratio (95% CI)	*p*
Age				
16–24	141 (47.8)	107/141 (75.9)	1	
25–30	86 (29.1)	60/86 (69.8)	0.73 (0.40–1.34)	0.07
30+	68 (23.0)	58/68 (85.3)	1.84 (0.85–4.00)	
Education				
Did not reach O Level	79 (26.4)	54/79 (68.4)	1	
Reached O Level	220 (73.6)	175/220 (79.5)	1.80 (1.01–3.20)	0.05
Food security				
Food secure	217 (73.1)	173/217 (79.7)	1	
Food insecure	80 (26.9)	54/80 (67.5)	0.53 (0.30–0.94)	0.03
Income				
Earns own income	90 (30.2)	64/90 (71.1)	1	
No personal income	208 (69.8)	164/208 (78.8)	1.51 (0.86–2.66)	0.15
Marital Status				
Married	267 (89.3)	212/267 (79.4)	1	
Divorced	20 (6.7)	7/20 (35.0)	0.14 (0.05–0.37)	< 0.001
Single& widowed	12 (4.0)	10 (83.3)	1.30 (0.28–6.09)	
Husband Income				
Earns income	245 (91.8)	196/245 (80.00)	1	
No income	22 (8.2)	16/22 (72.7)	0.67 (0.25–1.79)	0.42
Financial support				
No support	50 (16.7)	32/50 (64.0)	1	
Has support	249 (83.3)	197/249 (79.1)	2.13 (1.11–4.10)	0.02
Household income (continuous, unit = $10)			1.03 (1.01–1.05)	0.008
Number of children				
1	99 (33.1)	79/99 (79.8)	1	
2	91 (30.4)	70/91 (76.9)	0.84 (0.42–1.69)	^#^0.83
3	60 (20.1)	43/60 (71.7)	0.64 (0.30–1.35)	
4	31 (10.4)	23/31 (74.2)	0.73 (0.28–1.87)	
5+	18 (6.0)	14/18 (77.8)	0.89 (0.26–2.99)	
Whether any children died				
No	273 (91.3)	206/273 (75.5)	1	
Yes	26 (8.7)	23/26 (88.5)	2.49 (0.73–8.57)	0.15
History of miscarriage				
Yes	39 (13.1)	34/39 (87.2)	1	
No	259 (86.9)	194/259 (74.9)	0.44 (0.16–1.17)	0.1
Whether pregnancy was planned				
No	145 (48.5)	101/145 (69.7)	1	
Yes	154 (51.5)	128/154 (83.1)	2.14 (1.24–3.72)	0.007

^#^Score test for trend *p* = 0.42

Just under half of the women, 145 (48.5%), reported that their pregnancy was unintended. Of these, 74 (51.0%) had not been using contraception at the time of conception. Of the 71 women who were on contraception, 55 (77%) reported that they were using the contraceptive pill.

#### ANC registration and associated factors

Overall 229 women (76.6, 95%CI 72–81) registered for ANC. The mean gestation at time of registration was 29.5 weeks (95%CI 28.6–30.4). Table 2 shows the gestation at which women registered.

**Table 2:** Gestation weeks at time of ANC registration among 210 (91.7%) of those registered^a^

Gestation weeks at time of ANC registration	*N* (%)	Cumulative total	Cumulative %
5–8	2 (0.95)	2	0.95
9–12	3 (1.43)	5	2.38
13–16	6 (2.86)	11	5.24
17–20	12 (5.71)	23	10.95
21–24	16 (7.62)	39	18.57
25–28	39 (18.57)	78	37.14
29–32	65 (30.95)	143	68.10
33–36	45 (21.43)	188	89.52
37+	22 (10.48)	210	100.00

^a^Missing values for 8.3% of participants who registered

In univariable analysis, ANC registration decreased with factors that were measures of financial stress (Table 1). Using household income as a continuous variable, we found that the odds of registering for ANC increased by 3% for every $10 increase in household income, OR 1.03 (95%CI 1.01–1.05), *p* = 0.008). Out of 20 divorced women, seven (35%) registered for ANC, compared to 212 of 267 (79%) married women and 10 of 12 (83%) single and divorced women, *p* < 0.001. Higher education and having planned the pregnancy were associated with increased ANC registration.

In the final, adjusted model, the following variables remained associated with ANC registration: household income, marital status and education, which had a statistical interaction with whether the pregnancy was planned, Table 3.

**Table 3:** Multivariable analysis of factors associated with ANC registration

Factor	Adjusted odds ratio (95% CI)	*p* value
Household income	1.02 (1.0–1.04)^a^	0.07
Marital Status		
Married	1	
Divorced	0.20 (0.07–0.58)	0.003
Other (includes single and widowed women)	2.40 (0.48–11.98)	0.29
Ordinary level Education*planned pregnancy		
Did not reach ordinary level	1	
Reached ordinary level, did not plan pregnancy	0.75 (0.32–1.77)	0.51
Reached ordinary level, planned pregnancy	4.24 (1.70–10.55)	0.002
Whether pregnancy was planned*ordinary level education		
Did not plan pregnancy	1	
Planned pregnancy, did not reach ordinary level	0.58 (0.21–1.58)	0.28
Planned pregnancy, reached ordinary level education	3.27 (1.55–6.70)	0.002

^a^Odds ratio for a $10 increase in household income

### Qualitative study findings

Twenty-one participants with a median age of 26 years (range 16–38 years) were interviewed. Six were HIV positive; one did not know her status and the rest were negative. Most (17) were married. Only one third earned their own income. Sixteen had registered for ANC after week 24, with twelve registering in month eight, and two registering as late as 1 week before delivery. Four women had not sought ANC at all.

We identified generic barriers to ANC registration as well as those specific to provision of integrated HIV and PMTCT services, as summarised below.

#### User fees

All women reported that user fees were an important barrier to ANC registration. Discussion with participants revealed that their household incomes were so small that it was nearly impossible to save any money towards ANC registration.

*On a particular day my husband can bring home a meagre income of three dollars, on another day maybe he brings a mere five dollars…So it is quite a challenge to save up a substantial amount of money.* ***HIV positive participant***

Participants believed that if ANC fees were reduced or removed there would be better ANC uptake. Indeed, some participants reported that they had only managed to register when the clinic reduced the fees from $50 (the fee before the start of the study) to $30.

In this community where abject poverty is the norm, ANC registration competed with many basic household priorities. Women had to choose between saving up for ANC registration and other, sometimes critical or potentially life-altering alternatives. For example, women who became unwell during their pregnancy would further endanger themselves and their babies by failing to seek health care in order to protect the savings they had made towards the ANC fees. This is particularly sad because if they had already been registered at the onset of their illness, all their health care costs would have been covered.

*When you get here you are asked to pay five dollars for the card (consultation fee). After you have paid five dollars you will then buy medicines, okay. The total amount you pay can go up to ten or fifteen dollars. That fifteen dollars is a substantial amount of money; if you keep it diligently it can increase to thirty dollars (for ANC)* ***HIV positive participant***

### Feelings about non-registration: Anxiety due to exclusion

For women, not having paid the required ANC registration fees meant exclusion from the clinic. Women reported feeling helpless and “distressed” by this exclusion. Some worried about their own health while others worried about their baby’s welfare, including the risk of mother-to-child transmission in the context of unknown HIV status. It was clear that the majority of women appreciated the importance of ANC and wanted the reassurance that would come through ANC registration.

*I worried most about the baby; that it could possibly be in a breech position and I wouldn’t know about it. Or that the baby could die in the womb.* ***HIV negative participant***

Although in general participants displayed a strong appreciation of the importance of ANC, there were a few exceptions: three women reported that they had not been bothered by delaying their registration either because they already knew their HIV negative status or they felt they had a lot of time before delivery.

### Fear of HIV testing

The fear of HIV testing was frequently discussed as a barrier, although none of the women reported it as the reason she herself had not registered. Participants reported that ‘some women they knew’ did not seek ANC because they were afraid of discovering that they had HIV. There was the belief that knowledge of HIV positive status could result in that person “becoming stressed” as they would worry about their health. Another potential source of unease was the fear of stigma: a recurrent theme was that women worried about “how people would perceive them” if they discovered that they were HIV positive. The community was described in terms that suggested that HIV positive people were not accepted, and women reported worrying about losing or disrupting existing social relationships, particularly their marriage.

“*Now how do you tell your husband that you have been found to have the disease...maybe he can say I am no longer interested in you, and he just leaves you.”* ***HIV positive participant***

An “opt-out” testing policy was in place at the clinic, and women reported that ‘some women’ found it difficult to refuse testing. When registering for ANC, women were told to go for testing as a group; anyone who was unwilling to test remained sitting outside the clinic, which was socially awkward:

*What happens is when we are in a big group like that and it is called out, “It is now time for people to get tested,” one will think that if she refuses everyone will wonder what she is afraid of; “Oh my god, maybe she is HIV positive.”* ***HIV positive participant***

### Accounting for fear of HIV testing in the interviews

None of the women who talked about HIV testing as a barrier reported it as their own experience. Rather, it was described as the experience of other women, whom many participants reported to be “foolish”.

“*We could regard this as having a shallow mind because you could be harming your own and you could be killing yourself.”* ***HIV positive participant***

Thus it appeared that even if the woman may have been describing her own experiences she would consciously distance herself from her actions in order not to be associated with the image of a “foolish” woman who risked the health of her baby by not testing for HIV early.

### Nurse attitudes

As for fear of HIV testing, the role of identity creation [11, 12] in participant accounts was apparent: seven women reported that they knew other women who had not sought ANC because they wanted to avoid the clinic nurses who were reported to be discourteous towards clients. They then described instances where they felt the nurses had been discourteous towards them, in order to emphasise that this fear was justified.

*…the nurse was shouting at me saying, “You young people you rush to engage in sexual relations with men, this and that.”* ***Married participant***

Nurses were also feared not to uphold confidentiality of a woman’s HIV positive status.

### Unsupportive male partners

Eight women reported that they had faced partner opposition to ANC registration. Because most women were financially dependent on their husbands, they did not have the power to decide when they would register. Three reasons for the lack of male support were given: first, men did not appreciate the importance of the package of ANC; they reportedly thought it was only important in as far as it guaranteed an assisted delivery.

*He would say, “Why do you want to register the pregnancy when it’s still this small? You will register later isn’t it there is still plenty of time until you are nine months pregnant?”* ***HIV negative participant***

Second, the man’s assertion of authority reportedly affected ANC registration negatively.

*He will say, “I will tell you what to do. I will tell you when to do what. You will register when you are eight months pregnant.”* ***HIV negative participant***

Thirdly, the fear of their wife undergoing HIV testing reportedly played a role in men’s attitudes towards ANC registration.

“*Her husband hadn’t wanted her to register because he said she was going to have a blood test.”* ***HIV negative participant***

This gendered view is strengthened by the fact that mothers and mothers-in-law played an important role in getting the woman registered. Many women reported that it was their mother (or mother- in-law) that had brought about their eventual registration either by providing the ANC fees directly (mothers) or by pressuring their sons to give the registration money to the pregnant woman (mothers-in-law).

The role of mother-in-laws and the husband’s relatives in influencing the attitudes of male partners is also shown to be influential when we look at the cases where husbands were supportive. Of the three husbands reported as being supportive of ANC, two had been encouraged by their female relatives. In the other case his support of ANC appears to have been influenced by the desire for a HIV negative baby having undergone couples testing and counselling.

### Additional factors

Although not given as much emphasis by the women as the factors reported above, three other barriers were identified. The first was the long waiting times at the clinic. Six participants reported that waiting times at the ANC clinic were so long that it prevented women from registering, particularly women in self or formal employment who found it hard to take time off work.

*I would come. And I would find that there were many people at the clinic. So it became difficult for me to keep asking for time off from work. Then I didn’t do it.* ***HIV negative woman***

Second, five women reported that unintended pregnancies were not easy to register because they were discovered late in the gestation, which did not leave enough time to raise the ANC fees. One divorced woman said the emotional stress due to the divorce precluded thoughts about other things.

## Discussion

This study revealed sub-optimal ANC uptake among poor Zimbabwean women attending the largest polyclinic in Harare where ANC and PMTCT services are integrated: only 77% registered for ANC and they generally did this late in their pregnancy which affects not only their general antenatal care but also their ability to access timely and effective PMTCT. The survey found that educated women who had planned their pregnancy and those with higher household income were most likely to register. Divorced women were less likely to register than married women. The qualitative study provided more in-depth insight into the barriers to ANC uptake, revealing a connection between fear of HIV testing, unsupportive male partners and poor nurse attitudes, thus demonstrating general ANC barriers and those specific to integrated ANC and PMTCT services. Long waiting times and unintended pregnancies were also reported as barriers.

The ANC uptake rates in this study are considerably lower than other national estimates of 90–93% that were obtained at similar time points [13, 14]. This is likely explained by the extreme poverty of this group of women and the lack of free ANC services in their local community (in rural communities ANC is more likely to be offered without charge).

**Fig. 1:**
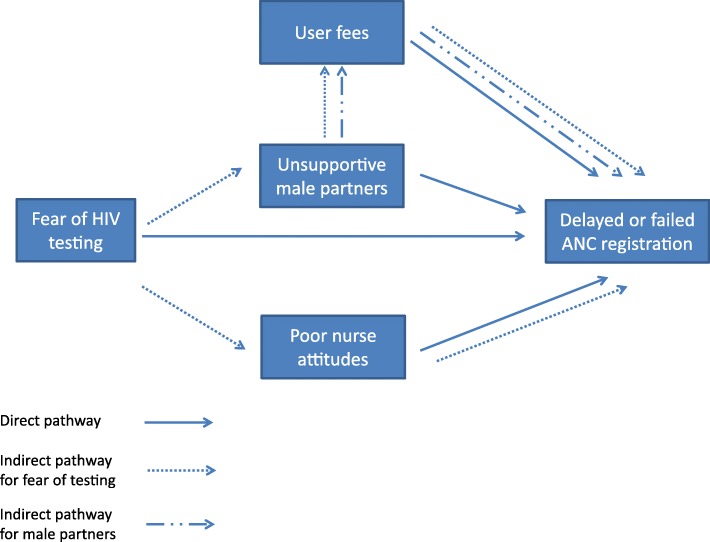
Conceptual framework

Based on the study results, we created a conceptual framework of general and HIV-specific barriers to antenatal care, indicating the additional barriers that come with integrating PMTCT into ANC services, Fig. 1. Although other frameworks for explaining barriers to PMTCT and skilled birth attendance have been developed [15, 16], this framework is unique in that it shows how the different barriers to service uptake interact in a setting where integrated services are provided. While integration is the model of choice for improving PMTCT outcomes, it comes at a cost of reducing ANC uptake among a subset of women who (or whose partners) are afraid of HIV testing. Health systems need to ensure that HIV tests are offered in a way, and in an environment, that 1) allows women to keep their decision to test or not confidential, and 2) makes it clear to women that they do not have to test for HIV in order to access ANC. This is not only important from a human rights perspective, but will ensure that women who do not want to get tested feel comfortable to seek ANC. Likely, if women who choose not to test are retained within the health care system, the continued education, counselling and support might enable them to take up HIV testing (although this should be done as soon as possible to ensure the baby’s protection from HIV). The effectiveness of such an approach in getting women to test may need to be tested using implementation science methods. Looking at male partners, the three instances where men were reported to be supportive of ANC suggest that interventions to promote couples testing and counselling and discussion of HIV testing among peers and family might help improve male support for ANC [17–19]. Providing male-friendly ANC clinic environments which support uptake of couples visits and HIV testing can be important. All this will be particularly important for the success of Option B+ (where women are initiated on lifelong ART during pregnancy or breastfeeding [20]), which is now being implemented in many countries, including Zimbabwe. It will also be important as countries begin scale up of the new WHO guidelines to treat all people diagnosed with HIV regardless of disease stage.

Health service-related factors (poor health care worker attitudes and long queues at the clinic) have also been reported in other studies [21, 7, 22]. It is critical that health workers are supported to provide better quality service. Results based financing or pay for performance programs have been found to improve service quality [23, 24].

The strength of this mixed-methods study was in the opportunity presented by the qualitative in-depth interviews in getting explanation of associations that were found in the survey. Qualitatively, user fees presented a formidable barrier to ANC and this offers explanation for the positive association between ANC registration and financial status, where it is conceivable that women who are more financially stable are better able to raise the required fees. The association of wealth and use of maternal health services has been reported in various settings [25–28]. In addition, removing/abolishing user fees has been found to positively impact uptake of health services in a number of countries [29–34]. However, lessons learned from those countries suggest that careful planning should be undertaken prior to abolition; it is important to forecast the impact this is likely to have on demand of health care services and resources such as drugs and human resources, followed by mobilisation of sufficient funds to support this process and ensure sustainability. The Zimbabwe Ministry of Health and Child Care policy is that user fees should be abolished but due to resource constraints this has not yet been adopted by the City Health Department [35]. Other interventions that can help women take up services include conditional and unconditional cash transfers [36, 37], and allowing women to stagger payments into smaller instalments which they can afford.

Given the difficulty women have in obtaining user fees, the qualitative findings highlight the importance of a pregnancy that is well planned to ensure sufficient time to raise the required fees, perhaps explaining the quantitative association of ANC registration with planned pregnancy and education. An educated woman who has planned her pregnancy is better able to think carefully about what her pregnancy entails so that she can mobilise the resources that are needed for ANC registration. This underlies the importance of ensuring both pre-conception education specifically and education of girls more generally. Importantly, about half of women in this study reported that their pregnancy was unintended, indeed half reported that they had been using contraception when they conceived emphasising the importance of strengthened reproductive health services and education.

The survey found that divorced women were significantly less likely to register for ANC when compared with married women. However, there are mixed findings from the literature where context and social structures may determine the association of marital status with use of maternal health services [28]. In our qualitative study, only one divorced/separated woman was interviewed in-depth, but her account may provide explanation to the observed association: where the divorced/separated partner is the father of the baby, the period during pregnancy may be filled with emotional stress as a result of the separation, which affects the woman’s ability to make other decisions that affect her life. For women who have been divorced for longer, there could be fear of social stigma to getting pregnant while divorced [28, 38], which may make them keen to hide their pregnancies for as long as possible. In addition, divorced women may register less possibly because they are less supported in their pregnancy, and they may have greater fear of having contracted HIV.

The main limitation of the survey was the low response rate of 54% - we only recruited women between Monday and Friday and during office hours. In addition, some women refused participation mainly because of time constraints. We do not have the number of women who refused participation, hence 54% is a conservative estimate which underestimates our actual response rate. Comparison of survey participants and general patient data collected as part of the wider study revealed similar characteristics which suggested that survey participants were representative of post-natal women at the clinic. Another limitation was recruitment of participants at the facility, which means that views of women who did not come to the clinic were excluded. However, just over 90% of Zimbabwean infants receive the BCG vaccine [14], which is given soon after delivery, pointing to high attendance of postnatal visits to the clinic, even by women who did not attend the clinic for ANC or delivery. At the time of the study, 33.5% of women delivered at home nationally [13]. In our study we surveyed 20 (60.8%) of participants who delivered at home. For the qualitative study, social desirability bias seemed to be at play during in-depth interviews where women may have worried about being perceived as ‘bad’ mothers.

Ideally, a qualitative study would have been conducted first in order to inform design of the quantitative survey, however limitations on time and budget did not allow for this type of design.

It has been long time since the study was conducted. Indeed, shifts in models of service provision have occurred since data were collected. However, the study findings on barriers to uptake of integrated services remain relevant not only for integrated maternal and HIV services, but for all integrated HIV services which we have now moved towards in the ‘treat all’ initiative.

## Conclusions

ANC uptake is sub-optimal particularly in poor communities where ANC fees are levied. We identified additional barriers to ANC that are a result of integration of PMTCT and ANC services. Interventions to increase ANC uptake in the context of PMTCT need to address general ANC barriers and those related to the fear of HIV testing. This is important for the success of Option B+ and ‘treat all’ initiatives.

## Additional file

Additional file 1: Cross sectional survey questionnaire. (PDF 835 kb)

## Abbreviations

ANC: Antenatal care
LMIC: Low and middle income countries
PMTCT: Prevention of mother-to-child transmission of HIV
WHO: World Health Organisation
