# Supplementary material for: “Well, not me, but other women do not register because...”- Barriers to seeking antenatal care in the context of prevention of mother-to-child transmission of HIV among Zimbabwean women: a mixed-methods study
Source: BMC Pregnancy Childbirth. 2018 Jun 28;18:271. doi: 10.1186/s12884-018-1898-7 (PMC6022348; doi:10.1186/s12884-018-1898-7)
Supplement: Supplementary file 1 — Cross sectional survey questionnaire. (PDF 835 kb) [file 12884_2018_1898_MOESM1_ESM.pdf]

ADDITIONAL FILE 1: QUESTIONNAIRE AT TIME OF DELIVERY

Patient questionnaire for completion at the time of delivery

Thank you for consenting to take part in this study that will enable us to have a better understanding of the experiences of expecting mothers in this community. I am asking you to give your responses to the questions which follow. Some questions may make you feel uncomfortable or distressed. Obviously if this happens we will stop the interview if you would like us to. As far as possible please give me your honest answers.

Date of interview: _______________ Clinic of delivery: ________________

DDMMMYYYY

Date baby was born: _______________

DDMMMYYYY

1. When were you born? /How old are you?
   Date of Birth: _________________ or current age__________________

   DDMMMYYYY

2. What is your religion?
   a. Roman Catholic
   b. Anglican
   c. Lutheran
   d. Methodist
   e. Baptist
   f. Presbyterian
   g. Apostolic
   h. Pentecostal
   i. Seventh Day Adventist
   j. Moslem
   k. African Traditional Religion
   l. Other (Specify)_____________
   m. No religion
3. What is the highest level of education that you have completed?
   a. None
   b. Primary school only
   c. Forms 1 to 2
   d. Forms 3 to 4
   e. Forms 5 to 6
   f. Diploma/Certificate/Degree
4. ***FOR RURAL PARTICIPANTS ONLY:*** Think about the building in your home that is the most appealing. What material is it built of?
   a. Pole and dagga
   b. Wood

   c. Mud bricks
   d. Cement blocks
   e. Stones
   f. Other (Specify): ______________________
      i. ***For Mbare Participants:***How many rooms does the house you live in have, excluding the toilet and bathroom?
         1. Specify:_______________
5. Are you staying here as?
   a. House owner
   b. Lodger
   c. Family member
   d. Other (Specify):__________________________
      i. How many people live in your household? ***Specify***: _________
6. What type of sewage disposal system do you use?
   a. Flush Bowl System
   b. Our own Blair toilet
   c. Neighbour's Blair toilet
   d. Bush
   e. Pit
   f. Other (Specify):______________
7. What source of drinking water do you use in your house? (choose one main source)
   a. Tapped communal
   b. Own tap
   c. Own borehole
   d. Communal borehole
   e. Unprotected well
   f. Protected well
   g. Stream/river
   h. Other (Specify)___________________
8. In the last week, has an adult in your house skipped a meal or eaten less in order for there to be enough food for the children?
   a. Yes
   b. No
9. In the last week, have you had to go an entire day without eating because there was no food in your household?
   a. Yes
   b. No
10. Approximately how much money do you earn/source each month in US dollars?
   a. I don't earn any money
   b. I earn $____________________
11. Are you currently married?
   a. Yes, I am currently married
   b. No, I am divorced ***go to 13***
   c. No, I am widowed ***go to 13***
   d. No, I have never been married ***go to 13***
12. Approximately how much money does your partner earn/source each month in US dollars?
   a. He does not earn any income
   b. He earns $____________________
13. Are you financially dependent on anyone?
   a. Yes
   b. No ***go to 14***

i. Who are you financially dependant on? ***Mark all that apply.***
   1. Husband/Partner
   2. Parents
   3. Mother/Father in law
   4. Brother/sister
   5. Bother/sister in law
   6. Someone else (Specify)________________________

14. How many children have you had in your life so far? (Specify)______________
15. Did any of your children die?
   a. Yes
   b. No ***go to 16***
      i. How many children died in total? Specify)_________________
      ii. How old was/were the child/children when they died? ***(Specify)***

Child 1=most recent death:

Child 1________years/months   Child 2_______years/months

Child 3________years/months

Other children who died:

____
____
____
____

For age greater than two years use years and months for age 24 months and younger

16. Now I am going to ask you about your children who are alive. We want to know how old they are and what their general health status is.

**Child**	**Age**	**Health Status**
		☐ Healthy<br>☐ Frequently ill
		☐ Healthy<br>☐ Frequently ill
		☐ Healthy<br>☐ Frequently ill
		☐ Healthy<br>☐ Frequently ill

Other children:

____
____

For age greater than two years use years and months for age 24 months and younger

Child 1=the most recent delivery ie the new born baby

17. Combined with question 16.

18. Have you had any miscarriages?
    a. Yes
    b. No ***go to 19***
        i. How many miscarriages have you had? ***(Specify)*___________**

        ii. How far gone with the pregnancy were you when you miscarried? ***Specify in number of weeks in each box as applicable***

Miscarriage 1	
Miscarriage 2	
Miscarriage 3	
Miscarriage 4	
Miscarriage 5	

Other miscarriages:

____

____

For some women being pregnant may be a joyful experience but for some it can be difficult.

19. For your last pregnancy, at what point did you discover that you were pregnant?
    a. In the first month
    b. In the second month
    c. In the third month
    d. More than three months into pregnancy
    e. I don't know
20. How did you discover that you were pregnant? ***Mark all that apply***
    a. I missed my monthly menstrual period
    b. Symptoms like nausea and vomiting (or other symptoms)
    c. I bought a pregnancy test kit and tested myself at home
    d. A health care worker gave me a pregnancy test or confirmed that I was pregnant
        i. What is the date of your last menstrual period? ***Interviewer to verify this date with the patient records***
            1. **Specify: ___ ___ /___ ___ ___ /___ ___ ___ ___**
               DDMMMYYYY

21. Did you plan to get pregnant?
    a. Yes
    b. No ***go to 22***
        i. Were you on a family planning method at the time you became pregnant?
            1. Yes
            2. No ***go to 22***

ii. What family planning method were you using?
   1. Pill
   2. Injectable contraceptive
   3. IUD
   4. Implant
   5. Other (Specify):____________________

22. How did you feel when you discovered that you were pregnant?
    a. Happy ***go to 23***
    b. Troubled
    c. Neither happy nor troubled ***go to 23***
       i. Why were you troubled? ***Mark all that apply***
          1. I was worried about the financial burden of having a new baby
          2. I just did not want to have another baby
          3. I was worried about my health
          4. I was worried about the health of the baby
          5. Other (Specify): ___________________________
23. Did you seek antenatal care after you discovered you were pregnant?
    a. Yes ***go to 24***
    b. No
       i. Why did you not seek antenatal care?
          1. I had no money
          2. I did not think it was important
          3. My husband/mother in law/other family member did not want me to
             (Specify the family member):__________________

          4. My religion does not allow me to
             Other: Specify________________________ ***to go 33***

24. Where did you seek antenatal care?
    a. Local clinic
       i. At this clinic (Name of clinic)
       ii. At another clinic (Specify)___________
    b. Local hospital
    c. Private doctor
    d. Other
       Specify: ______________________

25. When did you first seek antenatal care? Specify:_________________
    DDMMMYYYY

Interviewer to verify this date with the participant **records**

26. Deleted.
27. Did you keep visiting this health care centre (mentioned in 24a) after your first visit?
    a. Yes ***go to 28***
    b. No
       i. Did you visit another health care centre instead?
          1. Yes ***go to iii***
          2. No ***go to a***
             a. Why did you not have return clinic visits for antenatal care?
                i. I had no money

ii. I did not think it was important
iii. My husband/mother in law/other family member did not want me to
    1. Specify the family member:____________________
iv. My religion does not allow me to
v. Other:Specify_____________________

go to 30

ii. Deleted
iii. Why did you change health facilities?
    1. I had relocated
    2. I temporarily moved to be closer to my mother/another relative while I was pregnant
    3. I did not like the care I was getting at that clinic
    4. I could not afford the care I was getting at that health care facility
    5. Other (Specify):_______________________

28. Deleted.
29. Deleted.

It is common practise for women to get tested for some diseases or conditions, including sexually transmitted infections when they are pregnant. I would like you to tell me if you got tested for the following conditions.

30. Were you tested for high blood pressure? (Here we are talking about a procedure in which the health care worker places a cuff around your upper arm, applies pressure to it and then reads your bold pressure).
    a. Yes
    b. No ***go to 31***

DELETED 30 i-iv

31. Were you tested for high blood sugar level? (Here we are talking about a procedure in which the health care worker would have asked you to give a blood or urine sample that could be tested for diabetes).
    a. Yes
    b. No ***go to 32***

Deleted 31i-iv

32. Were you tested for syphilis? (here we are talking about a procedure in which the health care worker would have collected blood that would be sent for syphilis testing)
    a. Yes
    b. No ***go to 33***

    If yes

    i. At which month were you tested for syphilis?
        1. 1st Month
        2. 2nd Month
        3. 3rd Month
        4. 4th Month
        5. 5th Month
        6. 6th Month
        7. 7th Month
        8. 8th Month

9. 9th Month

ii. What was the outcome of the test?
  1. I tested negative for syphilis ***go to 33***
  2. I tested positive for syphilis
  3. I did not receive my results ***go to 33***

DELETED 33iii-vib

33. Have you been tested for HIV?
    a. Yes ***go to ii***
    b. No ***go to i***
       i. Why have you not been tested for HIV?
          1. I have been not offered the test ***go to 34***
          2. I refused the test ***go to 34***
          3. Other reason (Specify)__________________________ ***go to 34***
       ii. When were you tested for HIV? ***Mark all that apply***
          1. During 1st Month of last pregnancy (for your newest baby)
          2. During 2nd Month of last pregnancy, (for your newest baby)
          3. During 3rd Month of last pregnancy, (for your newest baby)
          4. During 4th Month of last pregnancy, (for your newest baby)
          5. During 5th Month of last pregnancy, (for your newest baby)
          6. During 6th Month of last pregnancy, (for your newest baby)
          7. During 7th Month of last pregnancy, (for your newest baby)
          8. During 8th Month of last pregnancy, (for your newest baby)
          9. During 9th Month of last pregnancy¸(for your newest baby)
          10. Before I got pregnant (last pregnancy)¸ (for your newest baby)
          11. I don't remember
       iii. Where was your most recent HIV test?
          1. Clinic
             a. This Clinic (Name of Clinic)
             b. Another Clinic (Specify)
          2. Hospital
          3. VCT Centre
          4. Other: Specify________________
       iv. What was the outcome of the test?
          1. I tested HIV positive
          2. I tested HIV negative ***go to 34***
          3. I did not receive my results
          4. I do not want to reveal my status to the interviewer ***go to 34***
       v. Are you taking antiretroviral drugs for the treatment of HIV? ***(interviewer please note this is different from the More Efficacious Regimen (MER) for PMTCT)***
          1. Yes
          2. No ***go to vi***
             a. When did you start taking the antiretroviral drugs?
                i. Before I got pregnant
                ii. When I was pregnant, before I was 8 months pregnant
                iii. When I was pregnant, after 8 months of pregnancy
                iv. I do not remember
             b. Do you take the antiretroviral drugs as prescribed?
                i. Never
                ii. Sometimes

iii. Most of the time
iv. All the time
Go to xii

vi. Did you receive the antiretroviral medicine AZT (Zidovudine) that you were to take twice daily from when you were about seven months pregnant?
1. Yes
2. No ***go to viii***
Check with Patient Medical Card

vii. Did you take the tablets twice daily as instructed?
1. Never ***go to a***
2. Sometimes ***go to a***
3. Most of the time ***go to a***
4. All the time ***go to viii***
a. What prevented you from taking them as instructed?
i. I forgot
ii. I lost the tablets
iii. My husband/mother in law or other relative did not allow me to (Specify)_____________
iv. My religion does not allow me
v. I was not able to collect some of my tablets from the clinic
vi. Other (specify)________________

viii. Did you receive nevirapine (medicine that reduces the chances of transmitting HIV to your baby) that you were supposed to take at the beginning of your labour pains?
1. Yes
2. No ***go to xi***

ix. Did you take the tablets as instructed?
1. Yes ***go to xii***
2. No

x. What prevented you from taking the tablets as instructed?
1. I forgot
2. I lost the tablets
3. My husband/mother in law or other relative did not allow me to (Specify)_____________
4. My religion does not allow me
5. I didn't want others to know that I was taking tablets
6. Other (Specify):________________ ***go to viii***

xi. Why did you not receive treatment?
1. I had no money
2. I refused the treatment
3. My husband/mother in law/ other family member prevented me from taking it ***(Specify which family member)***___________________
4. No treatment/medicine was available at the clinic
5. Other reason (Specify):_________________________

Interviewer to check the patient medical records to see what was prescribed to the baby and complete the following information.

Mark all that apply:

- i. ***Single dose nevirapine 2mg/kg***
- ii. ***AZT 4mg/kg 12 hourly for 7 days***
- iii. ***AZT 4mg/kg 12 hourly for 28 days***

xii. ***Interviewer to read as applicable***:

1. ***If baby was given any of the above medicines:*** I see on your card that your baby received medicine that helps prevent acquisition of HIV soon after delivery ***go to b***
2. ***If not given any medicine:*** I see on your card that your baby did not receive medicine that helps prevent acquisition of HIV soon after delivery ***go to xiii***
   - ***a. Removed.***
   - b. How many hours after you gave birth was the baby given medicine?
     - i. Within 24 hours
     - ii. Within 48 hours
     - iii. Within 72 hours
     - iv. I do not remember

     Go to xiv

xiii. Why did the baby not receive the medicine?

1. It was not offered
2. I refused
3. I do not know
4. Other (Specify)___________________

xiv. Did you tell anyone about your HIV status after you tested positive for HIV?

1. Yes
2. No ***go to 34***

xv. Who did you tell? ***Mark all that apply.***

1. Husband/partner
2. Friend
3. Mother
4. Sister
5. Brother
6. Aunt
7. Sister in Law
8. Other (Specify) __________________

xvi. Deleted.

34. Has your current or most recent partner been tested for HIV?

1. Yes
2. No ***go to 35***
3. I don't know ***go to 35***

ii. What was the result of the test?

1. Positive
2. Negative
3. He did not collect his result
4. He did not tell me

Now we would like to ask you about previous HIV tests you have taken.

35. Before this pregnancy, have you ever had any HIV tests?
    a. Yes
    b. No ***go to 36***
        i. How many times were you tested?
            1. Once
            2. Twice
            3. Three times
            4. Four times
            5. Five times
            6. More than five times
        ii. What motivated you to do the last HIV test before the last pregnancy? ***Mark all that apply***
            1. I was pregnant
            2. I was not feeling well
            3. I just wanted to know my HIV status
            4. There is someone/there are people who encouraged me to get tested
            5. It was a requirement for work/insurance (Specify)_________________
            6. My husband/partner and I decided to get tested before we got married or started having sex
            7. Other reason (Specify) _____________________
        iii. How much time had elapsed between the last HIV test you took (before this pregnancy) and the test you took during this pregnancy? ***Specify in number of years or months___________________***

        OR, IF NOT TESTED DURING LAST PREGNANCY:

        How long ago was the last HIV test you took?

        Specify in number of years___________________

        iv. What was the outcome of the last HIV test (before the one you took during this pregnancy?
            1. I tested HIV positive
            2. I tested HIV negative
            3. I did not receive my results
36. ***For patients who accessed ANC services ie answered yes to question 23:*** While you were still pregnant did you fall sick and visit the clinic between your scheduled antenatal visits to seek treatment?
    a. Yes ***go to ii***
    b. No ***go to 37 if HIV positive, otherwise go to 40***
        i. For those who did not seek antenatal care: While you were pregnant did you fall sick and visit the clinic to seek treatment?
        ii. How many times did you seek the medical care?
            1. Once
            2. Twice
            3. Three times
            4. Four times
            5. More than four times
            6. I do not remember

Now I want you to think about each visit you made during your last pregnancy in which you had come to seek treatment. For each visit we want to know what you were suffering from and how far gone in your pregnancy you were.

**Visit**	**What were you suffering from?**	**How far gone was your pregnancy?**
	☐ Headache<br>☐ Malaria<br>☐ Backache<br>☐ Stomach Problems<br>☐ Injury (accident, domestic violence, physical violence)<br>☐ General unwellness<br>☐ Other problem (specify):____________	☐ Less than 3 months pregnant<br>☐ Between 3 and 6 months pregnant<br>☐ More than 6 months pregnant
	☐ Headache<br>☐ Malaria<br>☐ Backache<br>☐ Stomach Problems<br>☐ Injury (accident, domestic violence, physical violence)<br>☐ General unwellness<br>☐ Other problem (specify):____________	☐ Less than 3 months pregnant<br>☐ Between 3 and 6 months pregnant<br>☐ More than 6 months pregnant
	☐ Headache<br>☐ Malaria<br>☐ Backache<br>☐ Stomach Problems<br>☐ Injury (accident, domestic violence, physical violence)<br>☐ General unwellness<br>☐ Other problem (specify):____________	☐ Less than 3 months pregnant<br>☐ Between 3 and 6 months pregnant<br>☐ More than 6 months pregnant
	☐ Headache<br>☐ Malaria<br>☐ Backache<br>☐ Stomach Problems<br>☐ Injury (accident, domestic violence, physical violence)<br>☐ General unwellness<br>☐ Other problem (specify):____________	☐ Less than 3 months pregnant<br>☐ Between 3 and 6 months pregnant<br>☐ More than 6 months pregnant
	☐ Headache<br>☐ Malaria<br>☐ Backache<br>☐ Stomach Problems<br>☐ Injury (accident, domestic violence, physical violence)<br>☐ General unwellness<br>☐ Other problem (specify):____________	☐ Less than 3 months pregnant<br>☐ Between 3 and 6 months pregnant<br>☐ More than 6 months pregnant
	☐ Headache<br>☐ Malaria<br>☐ Backache<br>☐ Stomach Problems	☐ Less than 3 months pregnant<br>☐ Between 3 and 6 months pregnant

	☐ Injury (accident, domestic violence, physical violence)<br>☐ General unwellness<br>☐ Other problem (specify):___________	☐ More than 6 months pregnant
	☐ Headache<br>☐ Malaria<br>☐ Backache<br>☐ Stomach Problems<br>☐ Injury (accident, domestic violence, physical violence)<br>☐ General unwellness<br>☐ Other problem (specify):___________	☐ Less than 3 months pregnant<br>☐ Between 3 and 6 months pregnant<br>☐ More than 6 months pregnant

iii. I am now going to ask you about tests that may have been carried out when you made visits to seek treatment. We want to know whether these tests were done.

1. Were you tested for high blood pressure? (Here we are talking about a procedure in which the health care worker places a cuff around your upper arm, applies pressure to it and then reads your bold pressure).
    a. Yes
    b. No
2. Were you tested for high blood sugar level? (Here we are talking about a procedure in which the health care worker would have asked you to give a blood or urine sample that could be tested for diabetes).
    a. Yes
    b. No
3. Were you tested for syphilis? (***here we are talking about a procedure in which the health care worker would have collected blood that would be sent for syphilis testing)***
    a. Yes
    b. No ***go to 37 if HIV positive, otherwise go to 40***
        i. At which month of your pregnancy were you tested for syphilis?
            1. 1st Month
            2. 2nd Month
            3. 3rd Month
            4. 4th Month
            5. 5th Month
            6. 6th Month
            7. 7th Month
            8. 8th Month
            9. 9th Month
        ii. What was the outcome of the test?
            1. I tested negative for syphilis ***go to 33***
            2. I tested positive for syphilis
            3. I did not receive my results ***go to 33***

Questions 37 to 39 only for those who are HIV positive

37. Have you been told where you can access HIV related care?

a. Yes
b. No ***go to 38***
    i. Have you been told when you should seek this care
        1. Yes
        2. No ***go to 38***
    ii. Do you think you are going to seek the care as advised?
        1. Yes ***go to 38***
        2. No
        3. I have already sought care ***go to 38***
    iii. Why do you think you are not going to seek the care?
        1. I do not have money
        2. My religion does not allow me
        3. My husband/mother-in-law/other relative will not allow me
            a. Specify which relative:________________
        4. People may stigmatise me if they see me seeking HIV related care
        5. Other reason

38. Do you think your baby is at risk of contracting HIV from you?
    a. Yes
    b. No
39. Have you been given any information about how to reduce your risks of transmitting HIV to your baby by the clinic staff?
    a. Yes
    b. No ***go to 40***
        i. What information were you given?

____

Are you planning to bring your baby to the six weeks baby clinic?

c. Yes ***end of form***
d. No
    i. Why do you not want to bring your baby to the six week visit?
        1. I do not think it is important
        2. It is too far to travel to the clinic
        3. I will go to another clinic
        4. It is too expensive to travel to the clinic
        5. I will be too busy to get time to come to the clinic
        6. My religion does not allow me
        7. My husband or mother-in law or other relative does not allow it (Specify) ____________
        8. Other reason (Specify)___________________

End of form
